# The cytosolic 5′-nucleotidase cN-II lowers the adaptability to glucose deprivation in human breast cancer cells

**DOI:** 10.18632/oncotarget.18653

**Published:** 2017-06-27

**Authors:** Gabriel Bricard, Octavia Cadassou, Laure-Estelle Cassagnes, Emeline Cros-Perrial, Léa Payen-Gay, Jean-Yves Puy, Isabelle Lefebvre-Tournier, Maria Grazia Tozzi, Charles Dumontet, Lars Petter Jordheim

**Affiliations:** ^1^ Université De Lyon, Université Claude Bernard Lyon 1, INSERM 1052, CNRS 5286, Centre Léon Bérard, Centre de Recherche en Cancérologie de Lyon, Lyon, France; ^2^ Biochemistry Laboratory of Lyon Sud, Hospices Civils de Lyon, Lyon, France; ^3^ IBMM, UMR 5247, CNRS - UM - ENSCM, Université de Montpellier, Montpellier, France; ^4^ Department of Biology, Biochemistry Unit, University of Pisa, Pisa, Italy

**Keywords:** 5’-nucleotidase, reactive oxygen species, glucose metabolism, hypoxia

## Abstract

The cytosolic 5’-nucleotidase cN-II is a highly conserved enzyme implicated in nucleotide metabolism. Based on recent observations suggesting additional roles not directly associated to its enzymatic activity, we studied human cancer cell models with basal or decreased cN-II expression. We developed cancer cells with stable inhibition of cN-II expression by transfection of shRNA-coding plasmids, and studied their biology. We show that human breast cancer cells MDA-MB-231 with decreased cN-II expression better adapt to the disappearance of glucose in growth medium under normoxic conditions than cells with a baseline expression level. This is associated with enhanced *in vivo* growth and a lower content of ROS in cells cultivated in absence of glucose due to more efficient mechanisms of elimination of ROS. Conversely, cells with low cN-II expression are more sensitive to glucose deprivation in hypoxic conditions. Overall, our results show that cN-II regulates the cellular response to glucose deprivation through a mechanism related to ROS metabolism and defence.

## INTRODUCTION

The hallmarks of cancer include genome instability and mutations, leading to deregulated energetic homeostasis, sustained proliferative signaling and escape from immune surveillance [1]. These major characteristics are all influenced by nucleoside metabolism as shown by: i) increased rate of genomic modifications when nucleotide pools are deregulated [2], ii) the important role of nucleotide derivatives as sources of energy for the cell and intracellular signaling effectors, and iii) the tumor- and immuno-modulating roles of adenosine and ATP [3, 4]. Therefore, nucleotide metabolism has become a subject of major interest in cancer research and constitutes a potential target for anticancer therapy.

5’-nucleotidases are involved in nucleotide metabolism by dephosphorylating nucleoside monophosphates into nucleosides and inorganic phosphate. There are today eight different human 5’-nucleotidases described, and they differ by their subcellular localization, substrate affinities and regulatory mechanisms [5, 6]. The cytosolic enzyme cN-II has a preference for IMP and GMP and has also been described as being capable of phosphorylating nucleosides through a phosphotransferase activity [7]. We have previously shown that this enzyme is involved in the sensitivity of cancer cells to nucleoside analogue-based chemotherapy [8, 9], and developed and studied enzymatic inhibitors [10–14]. The clinical relevance of this approach has been confirmed by the observation of hyperactive cN-II mutants in relapsed pediatric acute lymphoblastic leukemia patients associated with a resistance to purine analogues [15, 16]. However, very little is known about the overall physiological role of cN-II in cells, and especially in cancer cells from solid tumors. Transient inhibition of its expression in neuroblastoma cells by siRNA indicated a role in cell survival as this was associated with induction of apoptosis [17], whereas a similar decrease in skeletal muscle cells induced activation of AMPK (which regulates lipid and glucose metabolism) as well as modified lipid metabolism and glucose transport [18]. In addition, stable up- or down-regulation of cN-II expression in various cancer cells has shown its implication in cell proliferation even though this is not the case for all cell lines [9, 19, 20]. Finally, the recently demonstrated interaction between cN-II and the inflammasome-protein NLRC4/Ipaf suggests other and still unknown properties of this enzyme in cell biology that could be independent of its enzymatic activity [21].

In this study, we show that cN-II decreases the capacity to manage intracellular levels of reactive oxygen species (ROS), suggesting an important role of this protein in cell biology.

## RESULTS

### Transfected cells have decreased cN-II expression and enzymatic activity

The pScN-II cell models used in this study have previously been shown to have decreased cN-II protein expression [19]. This modification in protein expression was associated with a 1.3-2.2-fold decrease in specific enzymatic activity in all cell lines. Indeed, the specific enzymatic activity (nmol of inosine produced by minute per milligram of protein) in presence of ATP was 2.49 ± 0.20 for MDA-MB-231-pScont vs. 1.15 ± 0.04 for MDA-MB-231-pScN-II; 5.49 ± 0.75 for HCT-116-pScont vs. 2.89 ± 0.50 for HCT-116-pScN-II; 1.85 ± 0.25 for NCI-H292-pScont vs. 1.46 ± 0.16 for NCI-H292-pScN-II; 1.98 ± 0.14 for MIA PaCa-2-pScont vs. 1.33 ± 0.02 for MIA PaCa-2-pScN-II.

### Decreased cN-II expression is associated with enhanced *in vivo* xenograft growth

**Figure 1:**
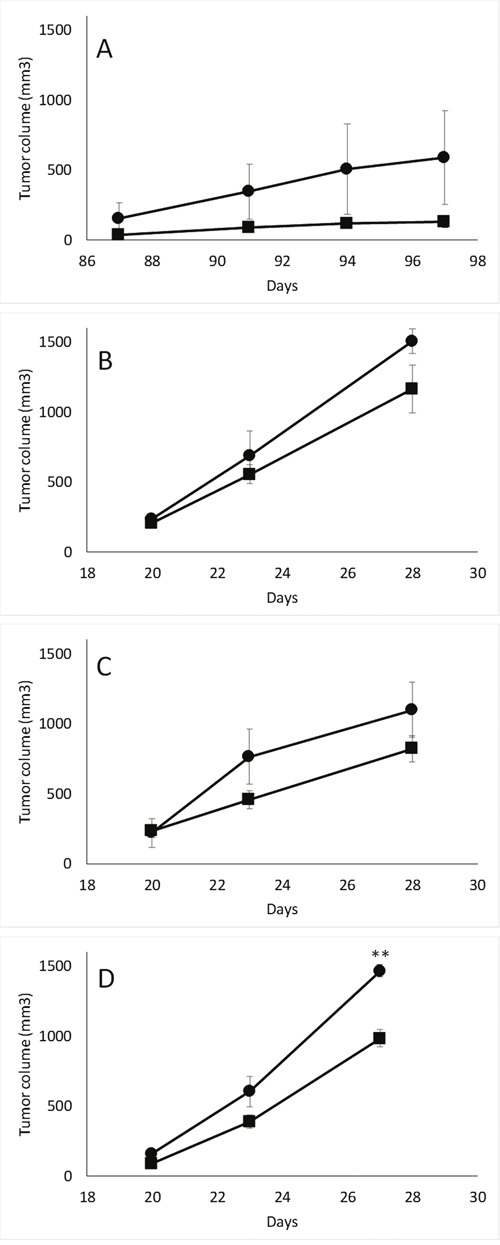
*In vivo* tumorigenesis of MDA-MB-231 **(A)**, HCT-116 **(B)**, NCI-H292 **(C)** and MIA PaCa-2 **(D)** pScont (■) and pScN-II cells (•). Tumor volumes are mean values from 3 mice per group and error bars are standard deviation. **: p<0.005 with Student's *t*-test.

Initial experiments of *in vitro* proliferation of the transfected models by CFSE titration did not show any differences between pScont and pScN-II cells [19]. We here continued the characterization with the evaluation of tumor growth in scid mice after the injection of 5 million cells subcutaneously. As indicated in Figure 1, the growth of pScN-II cells was consistently more rapid than for pScont cells in the four different models evaluated. This difference was modest and statistically significant for MIA PaCa-2 cells at day 27, suggesting that stably reduced content of cN-II in these cell models can favor tumor growth. Whereas tumors with NCI-H292, MIA PaCA-2 and HCT-116 cells reached a volume of approximately 1000 mm^3^ after 28 days, MDA-MB-231 cells grew more slowly.

### pScN-II cells have modified *in vitro* growth as compared to pScont cells

**Figure 2:**
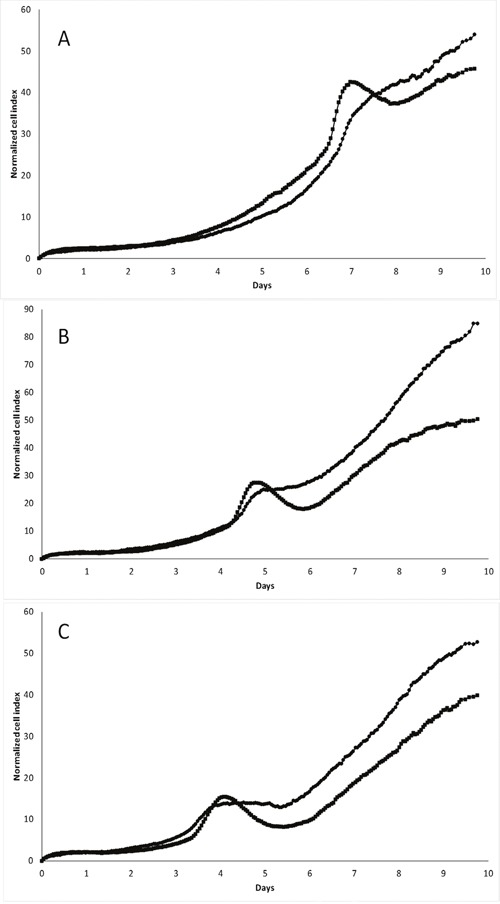
Long term *in vitro* cell growth of MDA-MB-231-pScont (■) and -pScN-II (•) cells in presence of 25 mM **(A)**, 10 mM **(B)** or 5 mM **(C)** glucose. Cells were seeded at 3000 cells per well in a final volume of 250 μl. Graphs show the normalized cell index during time (normalized on 5 hours).

To investigate the proliferation and behavior of the transfected cells *in vitro*, we performed long-term cell culture with real-time assessment of proliferation and adherence capacity using the xCELLigence technology. In these experiments, the cell culture media was not changed during the culture. As indicated in Figure 2 for MDA-MB-231 cells, there is a clear shift in the cell index appearing after approximately 7 days of culture in media containing initially 25 mM glucose. These variations in growth curves are much less pronounced for pScN-II cells as compared to pScont cells, indicating a major difference in the behavior between the two cell lines in response to the modifications appearing at this moment. When the initial concentrations of glucose in the media were lower, the same event appeared earlier (5 days with 10 mM, 4 days with 5 mM) and was always less pronounced for pScN-II cells. This suggested that the shift in cell index was associated with the disappearance of glucose in the culture medium. No interpretable growth curves were obtained with long term culture of any of the other cell models probably due to the rapid growth of these cells, and thus no glucose-dependent variations were observed. We therefore focused most of the further experiments on the MDA-MB-231 cell model.

### Decreased cN-II expression does not modify glucose uptake or lactate secretion *in vitro*

**Figure 3:**
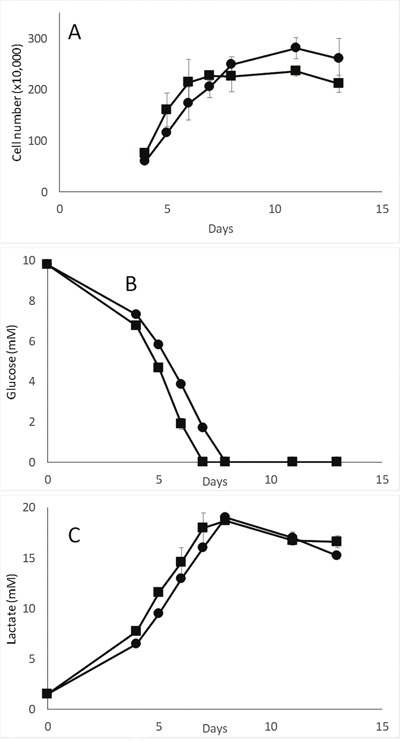
Cell number **(A)**, glucose **(B)** and lactate **(C)** concentration in media during long term *in vitro* culture of MDA-MB-231-pScont (■) and -pScN-II (•) cells. Cells were seeded in 6-well plates (90 000 cells per plate) in media containing 10 mM glucose. Values are mean results of duplicates from a representative experiment and error bars are standard deviation

As the MDA-MB-231-pScont and -pScN-II cells displayed different behavior in terms of cell index that was dependent on initial glucose concentration, we compared the glucose uptake and associated lactate secretion between the two cell models. When cells were seeded at the same ratio of cells/volume of media as in previous experiments and with 10 mM glucose, the glucose disappeared from the medium after 6 days of culture both for pScN-II and pScont cells (Figure 3). No notable differences in extracellular glucose concentration (reflecting glucose uptake) or in extracellular lactate concentrations (reflecting lactate secretion) were observed between the two cell lines when the cell number, as determined by direct counting, was taken into account. This suggested that the difference in cell behavior observed in Figure 2 was rather due to the ability of the different cells to adapt to culture media without glucose rather than to their use of glucose. In addition, as extracellular lactate levels reached 20 mM for an initial concentration of 10 mM glucose, it seems that the cells metabolized glucose preferentially through glycolysis rather than through oxidative phosphorylation.

### pScN-II cells have lower content of ROS during long-term *in vitro* growth

**Figure 4:**
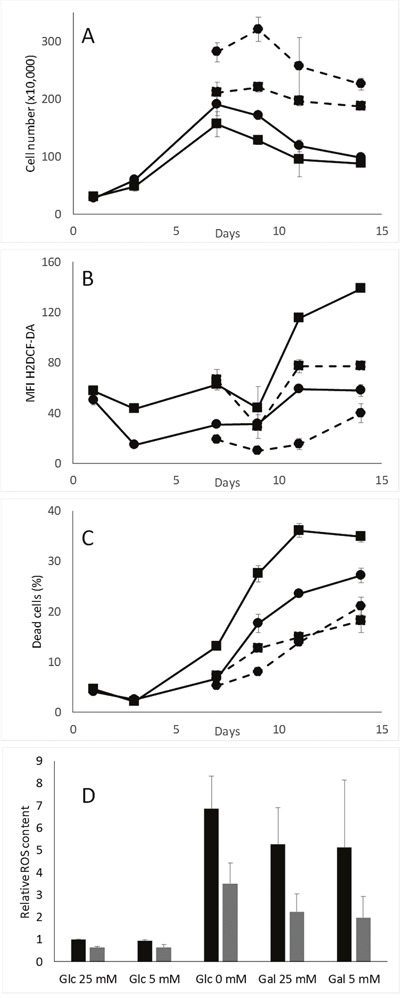
Cell number **(A)**, ROS content **(B)** and cell death **(C)** in MDA-MB-231-pScont (■) and -pScN-II (•) cells cultivated long-term without (full lines) or with renewed glucose (dotted lines). Cells were seeded in 6-well plates (180 000 cells per well in 3 mL of media with 5 mM glucose) and glucose (5 mM) was renewed three times a week. In A-C, results are means of duplicates from a representative experiment and error bars are standard deviation. **(D)** ROS content in MDA-MB-231-pScont (black) and -pScN-II (grey) cells cultivated for 3 days in media containing either 5 or 25 mM glucose or galactose or no sugar. Cells were seeded in 6-well plates (200 000 cells per well, 3 mL of media) and adhered overnight before culture in indicated media. In **(D)**, results are mean values of the ratio of MFI in each condition and the glucose 25 mM condition in three independent experiments and error bars are standard deviation. For both cell lines, Glc 0 mM and Gal 25 mM and Gal 5 mM were statistically significant (p<0.05, Student’s *t*-test) from Glc 25 mM, and for Glc 25 mM, Glc 5 mM, Glc 0 mM and Gal 25 mM differences between pScont and pScN-II were statistically significant (p<0.05, Student’s *t*-test).

When glucose is completely consumed, cells have to switch their metabolism towards the use of extracellular lactate as a carbon source or to beta-oxidation of fatty acids. Glutamine is another potential substrate but is highly unstable under our experimental conditions and is rapidly cleared from the culture medium. Lactate is transformed into pyruvate and acetyl-CoA while fatty acids release acetyl-CoA, which is further processed through the tricarboxylic acid cycle and oxidative phosphorylation in the mitochondrion. It has been shown that ROS-induced activation of AMPK further induces activation of pyruvate dehydrogenase kinase (PDK) and phosphorylation of pyruvate dehydrogenase (PDH) that stimulates lactate processing [22], and that AMPK stimulates beta-oxidation by ACC phosphorylation [23]. We propose that MDA-MB-231-pScN-II cells are more prone to perform this shift from glucose metabolism to lactate metabolism or to beta-oxidation. However, the oxidative phosphorylation is reported to be associated with enhanced levels of reactive oxygen species [24], which would rather be detrimental than beneficial for pScN-II cells. We therefore evaluated ROS levels in cells during cell culture simulating the conditions used during xCELLigence experiments. As shown in Figure 4A-4C, the ROS level increased in MDA-MB-231-pScont cells some days after the disappearance of glucose in the cell culture media (approximately when cell growth reaches a plateau), whereas ROS levels remained lower in pScN-II cells. The increase in ROS levels was associated with enhanced cell death as determined by Annexin V/PI staining, and both phenomena were delayed when glucose deprivation was avoided by adding 5 mM glucose to the media twice a week. A similar decrease in the ROS content was obtained by N-acetylcysteine instead of glucose during the experiment (data not shown). The influence of glucose starvation on ROS accumulation was confirmed in a 3-day experiment where pScont cells cultivated in absence of glucose accumulated much more ROS than pScN-II cells (Figure 4D). The replacement of glucose by galactose, which forces cells to perform oxidative phosphorylation, yielded similar results as for cells without glucose. Similar experiments performed on NCI-H292, MIA PaCa-2 and HCT-116 cell models did not show any differences between pScont and pScN-II cells (data not shown).

### Cells with low cN-II expression are sensitive to combined hypoxia and glucose deprivation

**Figure 5:**
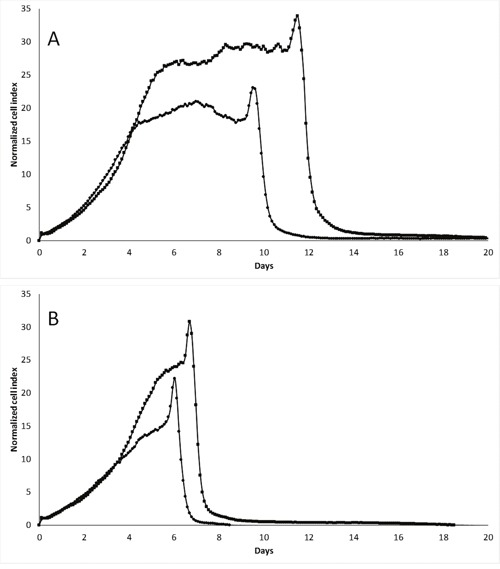
*In vitro* cell growth of MDA-MB-231-pScont (■) and -pScN-II (•) cells in hypoxic (1% O_2_) conditions in presence of 10 mM **(A)** or 5 mM **(B)** glucose. Cells were seeded at 3000 cells per well in a final volume of 250 μl. Graphs show the normalized cell index during time (normalized on 5 hours).

We further performed xCELLigence experiments in hypoxic conditions (1% O_2_), and observed that pScN-II cells were clearly more sensitive and died earlier than pScont cells (Figure 5). The time of cell death was here also dependent on the initial concentration of glucose in the culture medium suggesting that tolerance to reduced glucose was different under normoxic and hypoxic conditions.

**Figure 6:**
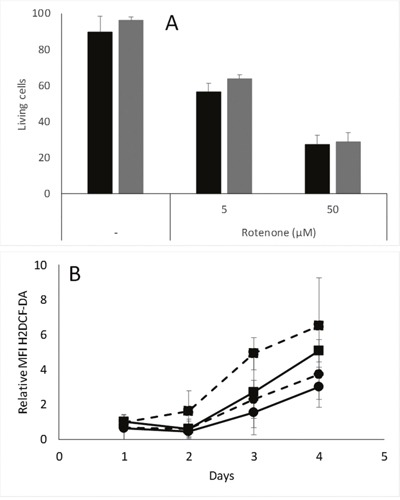
**(A)** Percentage of living MDA-MB-231-pScont (black bars) and -pScN-II (grey bars) cells incubated alone or in presence of indicated concentrations of rotenone and menadione. Cells were seeded in 6-well plates at 200 000 cells per well in 3 mL of culture media, and living cells are AnnexinV and propidium iodide negative. Results are mean values and error bars are standard deviation of three independent experiments. **(B)** Relative ROS content in MDA-MB-231-pScont (■) and -pScN-II (•) cells incubated with 1 mM glucose (filled lines) or without glucose (dotted lines) in hypoxic conditions (1% O_2_). Results are expressed as relative to MFI on day 1 for pScont cells incubated with glucose. Cells were seeded at 200 000 cells per well in 6-well plates with 3 mL culture media. Results are mean values and error bars are standard deviations of four independent experiments. Means were compared with Student’s *t*-test and differences were statistically significant at day 3 between pScont and pScN-II without glucose, and at day 4 between pScont and pScN-II with 1 mM glucose.

If the difference observed in cell survival in hypoxic conditions is due to a difference in remaining oxidative phosphorylation, the cells could have been expected to display different sensitivities to inhibitors of oxidative phosphorylation. This was however not the case in our MDA-MB-231 models, as the percentage of dead cells was similar in pScN-II cells as compared to pScont cells after 48 hour exposures to 5 or 50 μM rotenone, a mitochondrial complex I inhibitor (Figure 6A).

Finally, we evaluated the ROS content in pScont cells and pScN-II cells cultured in hypoxic conditions with or without glucose. We observed similar results as in normoxia, *i.e*. pScont cells accumulating higher levels of ROS than pScN-II cells in absence of glucose, and lower ROS contents for both cell lines when glucose was added to the medium during cell growth (Figure 6B). These results are consistent with the fact that pScN-II cells would have a lower induction of hypoxia-inducible factor-1 (HIF-1) as this is dependent both on hypoxia and on ROS content [25, 26]. Indeed, if pScN-II cells have less ROS in hypoxic conditions, they would have a lower induction of HIF-1, and thus a worse adaptability to the hypoxic condition as compared to pScont cells.

### cN-II downregulated cells have a better defense against ROS

**Figure 7:**
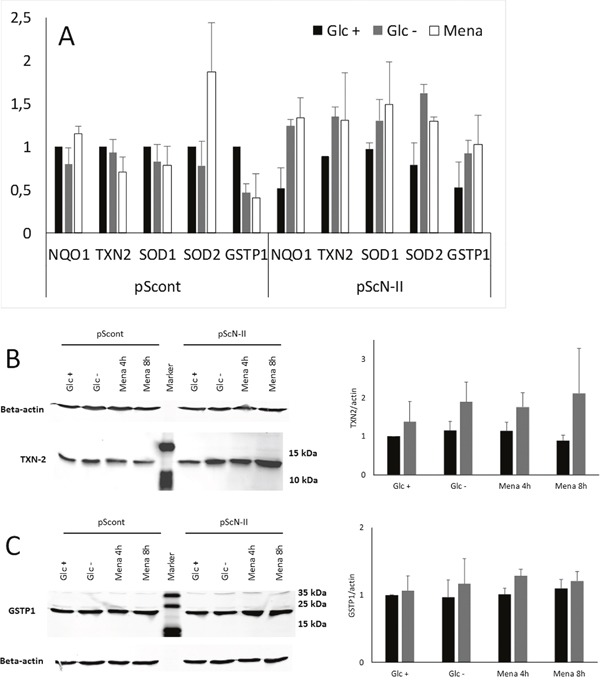
**(A)** Relative gene expression of anti-ROS genes in MDA-MB-231-pScont (left) and -pScN-II (right) cells incubated in presence (black bars) or in absence (grey bars) of 25 mM glucose, or in presence of glucose and 20 μM menadione (white bars) for 5 hours. Results are expressed as mean values of two independent experiments using pScont cells in presence of glucose as reference, and error bars are standard deviation. **(B** and **C)** Protein expression of TXN-2 **(B**, size 12 kDa) and GSTP1 **(C**, size 24 kDa) and beta-actin (size 42 kDa) in MDA-MB-231-pScont and -pScN-II cells cultured in presence (Glc +) or absence (Glc −) of glucose or in presence of glucose and 20 μM menadione (Mena) for 8 hours. The gels show results from a representative experiment and for the quantification, all samples were standardized to the ratio of protein/actin in pScont cells with glucose. The graph shows the mean values ± standard deviation of the quantification of bands obtained in three independent experiments. Means were compared with Student’s *t*-test but not found statistically significantly different.

As ROS levels were higher in pScont cells despite an apparently similar glucose consumption and metabolism, we assumed that the antioxidative defense mechanisms could be more abundant or more efficient in pScN-II cells. We first quantified the relative gene expression of NAD(P)H quinone dehydrogenase 1 (*NQO1*), thioredoxin-2 (*TXN2*), superoxide dismutase 1 and 2 (*SOD1* and *SOD2*) and glutathione S-transferase π (*GSTP1*). As shown in Figure 7A, these genes were expressed either at the same level or slightly less in pScN-II cells when cultured in presence of glucose. However, after 5 hours of culture in absence of glucose, these genes were all found to be more expressed in pScN-II cells as compared to pScont cells. Similar results were obtained when cells were exposed to the positive control menadione, a well-described ROS and anti-ROS defense inducer. This is in line with our hypothesis that pScN-II cells have a better overall capacity to respond to ROS after glucose deprivation.

This increase in gene expression was confirmed at the protein level for TXN2 but not for GSTP1 after an 8 hour incubation in the absence of glucose (Figure 7B). Indeed, still no difference was observed when cells were cultured in presence of glucose, but TXN2 expression increased 2-fold in pScN-II cells cultured in absence of glucose. Again, similar results were obtained with exposure to menadione.

### cN-II downregulation increases the autophagy flux

**Figure 8:**
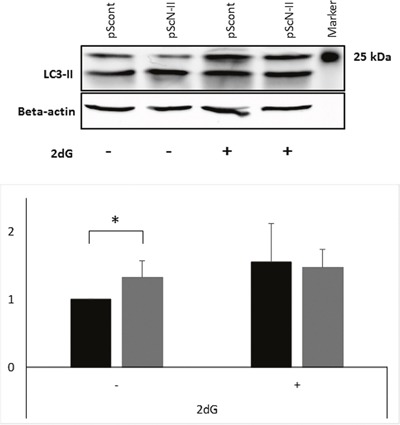
Protein expression of LC3-II (size 17 kDa) and beta-actin (size 42 kDa) in MDA-MB-231-pScont and -pScN-II cells cultured in normal media or exposed 16 hours to 2-deoxyglucose The gel shows result from a representative experiment and the graph shows the mean values ± standard deviation of the quantification of bands obtained in four independent experiments. *: p<0.005 with Student’s *t*-test.

Autophagy can be induced by energy deprivation, and contributes to the regeneration of ATP and other nutrients in the cells. Therefore, autophagy could also explain the metabolic advantages of pScN-II cells as compared to pScont cells. We investigated whether autophagy markers were differentially expressed between the two cell lines. As shown in Figure 8, pScN-II cells had higher expression of LC3-II, a marker for autophagy flux, than pScont cells when cultured in normal culture media. As expected, its expression increased after incubation with 25 mM of 2-deoxyglucose for 16 hours in pScont cells whereas this was not the case in pScN-II cells suggesting that the autophagy flux was already at its highest level in the basal conditions. It is however unclear whether autophagy is involved in the differences observed in hypoxic conditions.

## DISCUSSION

During our work on cN-II over the last 15 years, several observations have suggested that this enzyme might play major roles in human cells, independently from its activity in purine metabolism and sensitivity to nucleoside analogues [27]. First, transient inhibition by siRNA or by enzymatic inhibitors induce cell death in certain cancer cell models [12, 17]. Second, modulation of cN-II expression was associated with variations in cell growth rate and intracellular energy charge [20, 28]. Third, the highly conserved structure of cN-II among species, which is not limited to active and regulatory sites, suggested interactions with other cellular proteins [21]. Using our stable cell models for decreased cN-II expression, we here show consistent results on the implication of this enzyme in the regulation of cellular defense to oxidative stress.

Our results from the *in vivo* experiments show that the decrease of cN-II expression favors tumor growth. This is to our knowledge the first published data on tumor growth of cells with modified cN-II expression. However, this is not consistent with the previously reported results showing no difference in *in vitro* proliferation on these same cell lines [19] or decreased *in vitro* cell growth in a neuroblastoma model [20]. Enhanced tumor growth *in vivo* is thus not simply explained by enhanced proliferation of the cell lines in short term *in vitro* cultures. This difference in *in vivo* growth was however at least partially explained by our long term *in vitro* cell growth experiments. Our xCELLigence experiments were performed without renewal of culture media, and we showed that glucose was a limiting factor in this culture, since the differences in growth curves were observed earlier when cells were seeded in lower glucose concentrations (Figure 2). As this was not due to a difference in the glucose consumption between our cell lines, we proposed that the cN-II downregulation had induced a better capacity to respond to this low glucose environment. This could indeed be the case in the tumors as both cell lines grow similarly in the beginning, but diverge once they have reached a certain volume, eventually corresponding to a stage at which the tumor is poorly irrigated and the microenvironment is depleted of certain metabolites. However, such tumors would also be rather hypoxic, and thus more prone to cell death as suggested by our *in vitro* data. Unfortunately, due to technical issues, we were not able to obtain interpretable results on the xCELLigence apparatus for the glucose-dependency of NCI-H292, MIA-PaCa 2 and HCT-116 cells.

We also showed that the ROS content was much higher in control cells than in pScN-II cells when cells lacked glucose in the media either after long term culture or by incubating them directly without glucose. ROS-production by NADPH oxidase and mitochondrion has been shown to be induced by glucose deprivation in cancer cells and associated to subsequent cell death [29]. As, in our model, the highest ROS content was associated with a higher cell death, we conclude that this ROS content can directly explain the observed differences in cell death between our two cell lines. Furthermore, pScN-II cells displayed a better defense against ROS with both an enhanced induction of TXN2-expression and more autophagy.

One explanation to the observed glucose-deprivation-related differences between MDA-MB-231-pScont and -pScN-II cells could be the action of cN-II on metabolites of nicotinamide adenine dinucleotide (NAD). Indeed, it has been shown first in yeasts [30] and later in humans [31] that the mononucleotides of nicotinamide (NMN) and nicotinic acid (NAMN) are substrates for both cytosolic pyrimidine preferring 5’-nucleotidase (human cN-III and yeast Sdt1) and the purine preferring 5’-nucleotidase (human cN-II and yeast Isn1). Thus, it is possible that cN-II expressing cells have overall a lower level of NAD and its reduced form NADH (and thus of NADP) due to a higher degradation of their precursors, and consequently a poorer defense towards ROS that are produced in the presence of low glucose. The recent observation of an association between oxidative stress and an IMP/GMP-preferring 5’-nucleotidase (*yktC*) in *Bacillus subtilis*, strengthens this hypothesis [32].

When cells were grown under hypoxic conditions, the decrease of cN-II expression was shown to be associated with a higher sensitivity to the disappearance of glucose in the media. This could be explained by a higher ROS content in these cells. Indeed, it has been proposed that in hypoxic conditions, the ROS production is higher in cells with high NADH level [33]. This would again increase the glucose consumption by regulating GLUT-4 expression on the cell membrane as described in skeletal muscle cells [34]. This would be consistent with our hypothesis stating that pScN-II cells have a higher level of NADH because of a lower catabolism of its precursors. However, this was not the case in our ROS content quantification assays in cells grown in hypoxic conditions, suggesting another and yet unknown mechanism for this increased sensitivity to low glucose concentrations in hypoxia. It has been shown in a particular model that concomitant hypoxia and low glucose induced unfolded protein response [35]. This could indeed be the case also in our models, but additional experiments would be needed to confirm this.

The fact that pScN-II cells do not accumulate more ROS than pScont cells in hypoxic conditions whereas they die earlier (as shown by xCELLigence experiments in hypoxic conditions), could indicate that in normoxic conditions, a difference in ROS content is involved in the differential behavior between the two cell lines, whereas in hypoxic conditions, this difference is ROS-independent. We showed here that the pScN-II cells have a better overall anti-ROS defense, with a particular involvement of thioredoxin 2 and autophagy. The detailed mechanism by which these cells acquire this phenotype is not yet understood, but we believe that cells with downregulated cN-II expression will adapt through an increase of phosphorylated nicotinamide derivatives or through an adapted kinetics in AMPK activation. Ongoing studies should help us decipher this particular mechanism. Our results might have clinical relevance in settings where cancer patients are treated with metabolic inhibitors targeting directly or indirectly glucose metabolism. Indeed, such therapeutic approaches will be more efficient on cancer cells expressing high levels of cN-II than on cells with low levels of cN-II, making this protein a potential marker for response to treatment as already described in other clinical settings [8].

## MATERIALS AND METHODS

### Cells and culture

In this study we used human cancer cell lines from lung (NCI-H292), pancreas (MIA PaCa-2), colon (HCT-116) and breast (MDA-MB-231). Cells were transfected to express either a shRNA against cN-II (pScN-II cells) or control shRNA (pScont). General cell culture conditions and the development of transfected cell lines have been previously described [9, 19].

### cN-II activity assessment

cN-II activity in transfected models was assessed using a validated non-radioactive method of liquid chromatography coupled to a tandem mass spectrometry as described elsewhere [9].

### *In vivo* tumor growth

Female severe combined immunodeficiency CB17 mice (2-4 months old, approximately 20 g, Charles River, L’Arbresle, France) were injected with indicated amount of cells subcutaneously on day 1. Tumor size was measured twice a week and mice were euthanized when the tumor volume was >1500 mm^3^. The protocol for experiments in mice was approved by the University of Lyon Animal Ethics Committee. Mean tumor volumes were compared with Student’s *t*-test.

### Cell proliferation and adherence

The xCELLigence system (ACEA Biosciences) was used to concomitantly determine cell proliferation and adherence in real-time analysis. Cells were seeded (3000 cells per well, 250 μl of media) in 16-wells E-plates as indicated by the manufacturer and the cell index was recorded every 15 minutes for 24 hours then every 30 minutes up to 20 days. Cell proliferation and survival were also assessed by direct counting of cells both adhered to the flask and in the cell culture media using a Cellometer Auto T4 (Nexcelom Bioscience).

### Quantification of glucose and lactate in cell media

Cells were seeded in 6-well plates (90 000 cells per plate) in media containing 10 mM glucose and incubated at 37°C for indicated times. At each time point, cells were trypsinized and counted, and the supernatant was recovered for dosage of glucose and lactate. Glucose was quantified by the measurement of NADPH produced during enzymatic reactions, and lactate by its conversion to pyruvate and hydrogen peroxide followed by the conversion of ABTS into a chromogen as described earlier [36].

### Cell survival assay

Cell survival was assessed by the quantification of Annexin V/propidium iodide negative cells on a FACScalibur flow cytometer using Annexin-V-FLUO Staining kit (Roche) as indicated by the manufacturer and after culture in indicated conditions. Adherent and spontaneously detached cells were pooled for this analysis.

### Quantification of intracellular reactive oxygen species

The content of reactive oxygen species (ROS) in cells was determined using an oxidation sensitive fluorescent dye (H_2_DCFDA, Life Technologies). Cells were cultured as indicated and for ROS quantification, cells were washed with PBS, incubated 30 minutes at 37°C with 5 μM H_2_DCFDA, washed twice with PBS and incubated with complete media for 10 minutes at 37°C. Finally, cells were washed twice with PBS, trypsinized, centrifuged (5 minutes, 300 g) and resuspended in 200 μl PBS. ROS were measured by flow cytometry on a FACScalibur (λ_ex/em_: 490/530 nm) and compared to the autofluorescence of cells incubated with PBS instead of H_2_DCFDA.

### Western blot analyses

Proteins were extracted from cell pellets with cold RIPA buffer (20 mM Tris-HCl pH 7.5, 150 mM NaCl, 1% Triton X-100, 1% sodium deoxycholate, 1 M DTT, 1 M NaF, protease inhibitor cocktail, phosphatase inhibitors buffer and 100 mM sodium orthovanadate) or buffer A (20 mM Tris-HCl pH 6.8, 1 mM MgCl_2_, 2 mM EGTA, 0.5% NP40, 2% protease inhibitor cocktail) on ice for 60 (RIPA) or 15 (buffer A) minutes followed by centrifugation (15 minutes, 12 000 g, 4°C). Proteins were separated by SDS-PAGE and transferred onto nitrocellulose membrane using the iBlot® system (Life Technologies). Membranes were incubated with specific antibodies for LC3-II (NB100-2220, 1/500; Novus Biologicals), GSTP1 (A5691, 1/500; NeoBioLab), TXN2 (A6782, 1/500; NeoBioLab) and beta-actin (clone AC-15, 1/5000; Sigma) and anti-murine antibody (IRDye® 800CW, 1/5000; LI-COR Biosciences) or anti-rabbit antibody (IRDye® 680, 1/5000; LI-COR Biosciences), and protein expression was visualized using the Odyssey infrared system (LI-COR Biosciences). Bands were quantified using the Odyssey system, and the results are presented as ratio of the expression of proteins of interest to beta-actin expression.

### Quantitative RT-PCR

Total RNA was extracted from cells using RNeasy mini kit (Qiagen) as described by the manufacturer. Reverse transcription was performed with Moloney leukemia virus reverse transcriptase and quantitative PCR on a LightCycler thermal cycler (Roche) in the following conditions: 5 minutes initial denaturation at 95°C follow by 40 cycles of 10 seconds at 95°C, 10 seconds at 60°C and 10 seconds at 72°C and terminated by a melting curve from 70°C to 95°C. Primers were: NQO1 forward: 5’-ATGTATGACAAAGGACCCTTCC-3’; NQO1 reverse 5’-TCCCTTGCAGAGAGTACATGG-3’; TXN forward 5’-TTACAGCCGCTCGTCAGA-3’; TXN reverse 5’-AAGGCTTCCTGAAAAGCAGTC-3’; SOD1 forward 5’-TCATCAATTTCGAGCAGAAGG-3’; SOD1 reverse 5’-GCAGGCCTTCAGTCAGTCC-3’; SOD2 forward 5’-AAGTACCAGGAGGCGTTGG-3’; SOD2 reverse 5’-TGAACTTCAGTGCAGGCTGA-3’; GSTP1 forward 5’-GGCAACTGAAGCCTTTTGAG-3’ and GSTP1 reverse 5’-GGCTAGGACCTCATGGATCA-3’. Relative quantification was calculated using the ΔΔCT-method and human ribosomal 28S RNA as reference gene.

### Autophagy flux assessment

Cells (3.10^6^ per dish) were plated in 10 cm culture dishes and exposed or not to 25 mM 2-deoxyglucose (Sigma) for 16 hours before cells were subjected to protein extraction and Western blot analysis as indicated above.

## Abbreviations

pScN-II: pSuperior-cN-II
pScont: pSuperior-control
ROS: reactive oxygen species
NQO1: NAD(P)H quinone dehydrogenase 1
TXN2: thioredoxin-2
SOD1: superoxide dismutase 1
SOD2: superoxide dismutase 2
GSTP1: glutathione S-transferase π
